# Role of Monocyte Chemoattractant Protein-1 in Myocardial Infarction

**Published:** 2007-09

**Authors:** Hajime Morimoto, Masafumi Takahashi

**Affiliations:** 1 *Department of Cardiovascular Medicine and Regeneration, Matsumoto, Japan;*; 2 *Division of Cardiovascular Sciences, Department of Organ Regeneration, Shinshu University Graduate School of Medicine, Matsumoto, Japan*

**Keywords:** cytokines, inflammation, ischemia, leukocyte, myocardium, reperfusion

## Abstract

Myocardial infarction (MI) is accompanied by inflammatory responses that lead to the recruitment of leukocytes and subsequent myocardial damage, healing, and scar formation. Chemokines are a family of potent chemoattractant cytokines that regulate the leukocyte trafficking in basal levels and inflammatory processes; however, it has been recently recognized that chemokines are expressed by non-hematopoietic cells such as endothelial cells, smooth muscle cells, and cardiomyocytes, and their function extends far beyond leukocyte migration and activation. Many experimental and clinical studies have demonstrated that chemokines play an important role in the pathophysiology of MI. In particular, the CC chemokine – monocyte chemoattractant protein-1 (MCP-1/CCL2) – is one of the most frequently investigated, and it is believed to play an important role in the pathophysiology of MI. This review will focus on the role of MCP-1 in the pathophysiology of MI and discuss its potential as a therapeutic target in this condition.

## INTRODUCTION

Myocardial infarction (MI) is a common occurrence and it is predicted that this will be the leading cause of death worldwide in the near future. MI is defined as the necrosis of cardiovascular tissue due to ischemia that results in the replacement of the myocardium by a dense fibrotic scar. The major functional consequence of MI is a decrease in systolic compliance, i.e., progressive loss of pump function in the chamber where the loss of muscle had occurred. In addition, MI often results in electrical instability within the heart and generation of fatal arrhythmias. Therefore, the development of MI may be an important issue that needs to be studied in the 21^st^ century.

The most common cause of MI is atherosclerosis in the coronary arteries (1). The atherosclerotic plaques may become unstable, may rupture, and facilitate the formation of a thrombus that occludes the coronary artery; this leads to MI. In humans, myocardial necrosis begins in the subendocardium at 30–40 minutes after the onset of coronary occlusion: an almost complete infarction of the area at risk develops after approximately 4 hours of coronary occlusion. However, the exact time for complete infarction is influenced by several other factors including collateral circulation, prior ischemic events, and neurologic reflexes. Furthermore, increased fibrinolytic activity may lead to restoration of the coronary blood flow. In such cases, coronary blood flow is restored and causes “reperfusion injury”. On MI occurrence, the infarcted myocardium is injured by the ischemic insult and this result in myocardial cell death, i.e., necrosis and apoptosis. Simultaneously, circulating leukocytes are recruited to the site of the infarcted area, and subsequently, healing and scar formation occurs. The ischemic insult begins immediately after the coronary occlusion that lasts for several hours: subsequent leukocyte recruitment is also observed. Myocardial healing and myocardial scar formation begins at approximately 72 hours and 1 week, respectively, after the onset of the ischemic insult. In addition, concomitant angiogenesis might influence the degree of myocardial damage and remodeling after MI.

Recent evidence indicates that MI is accompanied by inflammatory responses that lead to the recruitment of leukocytes and subsequent myocardial damage, healing, and scar formation (2). Furthermore, the leukocytes recruited to the site of the infarcted myocardium lead to the release of cytokines and proteinases that may induce further inflammation and left ventricular remodeling. These cells also secrete a large amount of angiogenic factors such as the vascular endothelial growth factor (VEGF) that can induce angiogenesis in the infarcted heart. Thus, inflammation plays a role in the pathological processes from the initiation to the end of MI. These processes include: myocardial necrosis and apoptosis, leukocyte recruitment, myocardial healing and scar formation, and angiogenesis.

Chemokines are a family of potent chemoattractant cytokines that regulate leukocyte trafficking in basal levels and inflammatory processes (3, 4). Depending on its topical concentration, they directly recruit circulating leukocytes to the site of inflammation or injury. Many experimental and clinical studies have demonstrated that a substantial number of chemokines are involved in the pathophysiology of MI. Among these, monocyte chemoattractant protein-1 (MCP-1, also known as CCL2) is one of the most frequently investigated, and it is believed to play an important role in the process of MI. Indeed, during a 10-months follow-up in a large study involving patients with acute coronary syndromes, elevated serum MCP-1 levels were shown to be associated with an increased risk of death or MI (5). Furthermore, a recent study demonstrated that MCP-1 gene polymorphisms are associated with increased serum MCP-1 levels and prevalent MI in the Framingham Heart Study (6). Since MCP-1 mainly recruits monocytes to the site of inflammation and the infarcted area (7), it may play a role in the early stages of atherosclerosis or in the subacute phase of MI. Recently, other investigators and we demonstrated that MCP-1 plays a significant role in the process of myocardial healing and remodeling after MI occurrence (8-11). This review will focus on the role of MCP-1 in the pathophysiology of MI and discuss the potential of MCP-1 as a therapeutic target in MI.

## CHEMOKINES

It has been demonstrated that a number of factors and molecules are involved in the pathophysiology of MI. Among these, chemokines are thought to be one of the key regulators because they have not only been shown to be expressed in the infarcted myocardium but they also play a crucial role in the process of myocardial inflammation and healing (12, 13). In humans, more than 50 chemokines have been identified and divided into two main subfamilies on the basis of the conserved structural features. In the CXC chemokines, a single amino acid separates the two amino–terminal cysteine residues; however, in the CC chemokines, no amino acid separates the two cysteines. Other minor chemokine subfamilies that are currently known include fractalkine/CX3CL1 (CX3C chemokines) and lymphotactin/XCL1. Generally, CC chemokines are potent chemoattractants and activators of monocytes and lymphocytes, whereas CXC chemokines attract neutrophils.

Chemokines mediate their effects via interaction with specific chemokines receptors that are expressed on a wide range of cell types. The chemokine receptors are seven transmembrane G-protein-coupled receptors, and are unusual among the many characterized members of the seven transmembrane receptor superfamily in that a single receptor possesses multiple high-affinity ligands for it. Therefore, initially, the chemokine network might appear like a redundant cellular signaling system; however, analysis of chemokine or its receptor gene disruption in mice has revealed that certain chemokines possess unique and non-redundant roles in leukocyte trafficking, inflammation, and immunity.

The basic role of chemokines involves regulation of leukocyte transport and trafficking in basal and inflammatory processes; however, recently, it has been observed that chemokines are also expressed by non-hematopoietic cells such as endothelial cells, smooth muscle cells, and cardiomyocytes, and their function extends far beyond leukocyte migration and activation. For example, the CXC chemokines exert angiogenic or angiostatic effects on endothelial cells. Strieter *et al*. (14) reported that CXC chemokines containing the ELR (glutamate-leucine-arginine) motif such as interleukin-8 (IL-8, known as CXCL8) stimulate the migration of endothelial cells and promote angiogenesis. In contrast, other CXC chemokines such as platelet factor 4 (PF-4, known as CXCL4) and interferon-inducible protein 10 (IP-10, known as CXCL10) fail to induce endothelial migration or angiogenesis but exert angiostatic effects in the presence of other angiogenic factors (14). Thus, chemokines are key mediators not only in inflammatory responses but also in other responses in the pathophysiology of diseases.

### Chemokines involved in myocardial infarction

Many experimental and clinical studies have shown that chemokines are involved in the pathophysiology of MI. The CXC chemokines IL-8 (15-18), stromal cell-derived factor-1 (SDF-1, known as CXCL12) (19-21), GRO-α/KC (known as CXCL1) (16), and IP-10, and the CC chemokines MCP-1 (7, 9, 15, 22-25) and macrophage inflammatory protein-1α/β (MIP-1α/β, known as CCL3/4) (23, 26) appear to be consistently up-regulated in various animal models of experimental MI (Table 1). Among these, two types of MI models are currently used, i.e., permanent MI and ischemia-reperfusion injury models. Actually, several differences exist between the pathophysiology of permanent MI and ischemia-reperfusion injury (27, 28). Reperfusion releases a large excess of oxygen-derived free radicals and causes “reperfusion injury”. Inflammatory responses such as the infiltration of neutrophils and macrophages are much stronger in the reperfused heart than in the infarcted heart. Since the inflammatory responses after MI determine for tissue healing (2), collagen deposition during MI may be accelerated in the reperfused heart. Furthermore, reperfusion enhances neovascularization to a greater extent in the reperfused heart than in the infracted heart. Therefore, it is necessary to pay attention to the MI models used in the studies. Consistent with the results of the experimental studies, elevated levels of MCP-1 were detected in the sera obtained from MI patients (Table 2) (29-34); this indicates the clinical significance of these chemokines in the pathophysiology of MI.

**Table 1:** Chemokines in experimental MI models

Model	Species	Chemokine	Detection	Expression site	Time (**Peak**)	References

AMI	Mouse	MCP-1	RPA	Non-infarct	Day 1, **3**, 7, 28	9
AMI	Mouse	SDF-1	PCR ELISA	Infarcted myocardium	48, **72**, 96 h	19
AMI	Mouse	SDF-1	RT-PCR WB	Infarcted heart	0.5, **1**, 2, 4, 8, 16 day	20
AMI	Rat	SDF-1a	NB	Left ventricle	0.25h, 1h, 24h, **1w**, 3w, 6 week	21
AMI	Rat	MCP-1f IL-8	NB	Infarcted area	**1**, 6 week	15
AMI	Rat	IL-8 GRO-α	TR-PCR ELISA	Ischemic heart Serum	**6**, 12, 24, 48 h	16
AMI	Rat	MCP-1	ISH IHC	Infarcted area	Day 3, **7**, 14, 21	24
I/R	Mouse	MIP-1α MIP-1β MIP-2	RPA IHC	Myocardium	15 min I + 3hR	26
I/R	Mouse	MCP-1 MIP-1α MIP-2	RT-PCR	Ischemic heart	30 min I + 120 min R	23
I/R	Rat	MCP-1	NB	Ischemic reperfused myocardium	25 min I + 2 h R	22
I/R	Rabbit	IL-8	RIA	Ischemic myocardium	45 min I + 1.5, 3, **4.5** h R	17
I/R	Rabbit	IL-8	ELISA	Ischemic heart	1, 2, **4**, 8 h	18
I/R	Dog	MCP-1	NB ISH	Reperfused area	1, **3**, 5, 24, 48 h	7
I/R	Dog	MCP-1	RPA ISH	Ischemic area	15min I + 5 h R	25

AMI, acute myocardial infarction; I, ischemia; R, reperfusion; RPA, ribonuclease protection assay; PCR, polymerase chain reaction; RTPCR, reverse transcriptase-PCR; WB, western blot; NB, northern blot; ELISA, enzyme-linked immunosorbent assay; ISH, *in situ* hybridyzation; IHC, immunohistochemitry; RIA, raioimmunoassay.

**Table 2:** MCP-1 in MI patients

Sample collection	Detection	References

Plasma about 2 weeks after the MI	ELISA	29
onset MCP-1: 932 ± 193 pg/mL (n=16)		
Serum on admission	ELISA	30
MCP-1: 135.9 12.5 pg/mL (n=12)		
Plasma	ELISA	31
MCP-1: 191.5 pg/mL (n=30)		
Pericardial fluid within 7 days of the MI onset	EIA	32
MCP-1: 190 ± 49 pg/mL (n=16)		
Plasma	EIA	33
MCP-1: 268 ± 34 pg/mL (n=14)		
Serum within 24h after the MI onset	ELISA	34
MCP-1: 360 pg/mL (n=64)		

MI, myocardial infarction; ELISA, enzyme-linked immunosorbent assay; EIA, enzyme immunoassay.

Since CXC chemokines predominantly recruit neutrophils to the infarcted myocardium, they may play a role in myocardial injury after ischemia-reperfusion. With regard to IL-8, recombinant IL-8 markedly increased the adhesion of neutrophils to isolated cardiomyocytes, resulting in direct cytotoxicity for the cells (35); this suggests a potential role in neutrophil-mediated myocardial injury. Furthermore, neutralization of IL-8 significantly reduces the degree of necrosis in a rabbit model of myocardial ischemia-reperfusion injury (36). Therefore, IL-8 is assumed to be deleterious for MI although it has angiogenic effects. Another reported CXC chemokine SDF-1 and its receptor CXCR4 play a critical role in cardiovascular development and angiogenesis (37, 38). We recently found that SDF-1 is markedly upregulated in the infarcted heart, and that CXCR4 cells mobilized by the macrophage colony-stimulating factor (M-CSF) are recruited to the infarcted myocardium, resulting in improved cardiac dysfunction and remodeling after MI (39). In support of this evidence, very recently, Misao *et al*. (40) reported that the SDF-1-CXCR4 axis contributes to the beneficial effects of granulocyte colony-stimulating factor (G-CSF) in a rabbit model of myocardial ischemia-reperfusion injury. The expression of GRO-α/KC, lipopolysaccharide-induced CXC chemokine (LIX, known as CXCL5), and IP-10 was shown to have increased after MI (41, 42); however, the precise role of these CXC chemokines in MI remains unclear. On the other hand, since CC chemokines mainly recruit monocytes and lymphocytes to the infracted myocardium, they may play a role in the process of healing and scar formation after MI. MIP-1α/β is also upregulated in the infarcted myocardium (26); however, its role remains to be elucidated.

The mechanisms responsible for chemokine upregulation in the infarcted heart have not been fully understood; however, the factors implicated in initiating the inflammatory responses are likely to stimulate the induction of these chemokines. IL-8 and MCP-1 induction is regulated by a transcription factor NF-κB, which contributes to the regulation of inflammation. Recently, it was shown in rats and mice that NF-κβ is activated in the infarcted heart and that its inactivation improves cardiac dysfunction and remodeling after MI (43, 44); this suggests the involvement of NF-κB in the pathophysiology of MI. Since inflammatory cytokines such as tumor necrosis factor-α (TNF-α) and interleukin-1β (IL-1β), and reactive oxygen species (ROS) activate NF-κB in cardiomyocytes, these factors may contribute to NF-κB activation in the infarcted myocardium. SDF-1 induction is regulated by another transcription factor, i.e., hypoxia-inducible factor-1α (HIF-1α), which was induced by hypoxia (45). HIF-1 was also reported to be in the active form in the infarcted myocardium after MI (46).

## MCP-1 IN MYOCARDIAL INFARCTION

**Figure 1:**
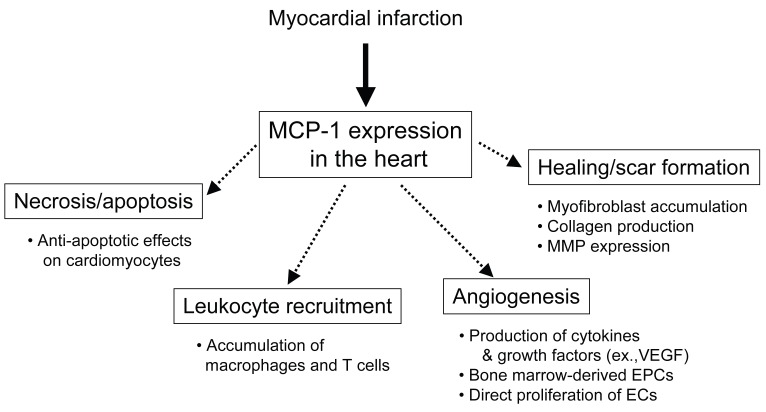
The role of MCP-1 in pathophysiology of MI. MCP-1 may affect many processes involved in MI; myocardial necrosis and apoptosis, leukocyte recruitment, myocardial healing and scar formation, and angiogenesis.

MCP-1 is one of the best-studied CC chemokines that is involved in pathophysiology of MI. MCP-1 induces the recruitment and activation of monocytes, T cells, and NK cells but not neutrophils; it has been implicated in diseases characterized by monocyte-rich infiltrates. MCP-1 is a major ligand for the CC chemokine receptor 2 (CCR2) and binds only to CCR2. CCR2 is one of CC chemokine receptors that are G-protein-coupled receptors with seven transmembrane domains (47). Activation of CCR2 by MCP-1 induces its dimerization, internalization, and activation of downstream signaling pathways including G-proteins, JAK/STAT, mitogen-activated protein (MAP) kinase pathways (48-50); however, little is known about the mechanism of MCP-1 signal transduction via CCR2 in the myocardium. MCP-1 is produced by a variety of cells in response to injury or exposure to other cytokines, such as IL-1β, IL-6, and TNF-α, and has been implicated in heart failure, ischemia-reperfusion, and MI (13, 51). Several transcription factors have been proposed for the regulation of MCP-1. In human MCP-1 gene, tissue-type plasminogen activator (t-PA)-responsive elements (TRE) and κB enhancer element exist at the 5’-flanking region of MCP-1 gene; this suggest that the role of activator protein-1 (AP-1) and NF-κB (52). Further, several signaling molecules are involved in the activation of MCP-1 gene, including protein kinase C and tyrosine kinases (53). Recent evidence suggests that MCP-1 may affect many processes involved in MI, namely, myocardial necrosis and apoptosis, leukocyte recruitment, myocardial healing and scar formation, and angiogenesis (Fig. 1).

### Myocardial necrosis and apoptosis

In acute MI, a large number of cardiomyocytes die as a result of apoptosis and necrosis (54-56). Recent studies suggest that ischemia-reperfusion, not ischemia alone, may be required to complete the apoptotic process in cardiomyocytes. Zhao *et al*. (56) discovered that prolonged ischemia without reperfusion induces necrosis but not apoptosis, whereas ischemia followed by reperfusion induces apoptosis. Another report confirmed that the apoptotic process is triggered by ischemia and reperfusion is required to complete this process (55).

Since monocyte/macrophage recruitment to the infarcted myocardium was observed in the subacute phase of MI, it is unlikely that the monocyte chemoattractant effects of MCP-1 contribute to this process. However, recent investigations suggest that MCP-1 affects other types of cells such as cardiomyocytes and endothelial cells (57-60). In particular, Tarzami *et al*. (60, 61) recently tested the effect of MCP-1 on apoptosis induced by hypoxia in neonatal rat cultured cardiomyocytes and reported that MCP-1 protects the cardiomyocytes from hypoxia-induced apoptosis. The anti-apoptotic effect of MCP-1 is mediated via several pathways: extracellular-regulated kinase 1/2 (ERK1/2) and Bcl-2 family proteins, but not Gαi. Interestingly, the monocyte chemoattractant effect is mediated by the activation of the G protein Gαi (62); this process is inhibited by the pertussis toxin. Therefore, anti-apoptotic and monocyte chemoattractant effects of MCP-1 may be mediated via the different signaling pathways. The *in vivo* role of MCP-1 in apoptosis and/or necrosis in the infarcted heart is currently unclear.

### Leukocyte recruitment

Since MCP-1 is a potent chemoattractant for monocytes, MCP-1 plays a substantial role in the recruitment of monocytes/macrophages to the infarcted myocardium. Since the chemoattractant effect of MCP-1 is regulated by its topical concentrations (63, 64), we investigated the role of cardiac MCP-1 that is topically produced in the infarcted heart; transgenic mice expressing the mouse *JE*-MCP-1 gene under the control of the α-cardiac myosin heavy chain promoter (MHC/MCP-1 mice) were used for this purpose (8). At 12 weeks, the MHC/MCP-1 mice showed a slight increased in macrophage infiltration in the heart; however, no cardiac dysfunction and hypertrophy were observed. After MI, the macrophage infiltration increased in the infarcted area of the heart in wild-type mice and it markedly increased in MHC/MCP-1 mice. Concomitantly, capillary formation is also promoted in the infarcted heart of MHC/MCP-1 mice. Hayashidani *et al*. (9) reported that anti-MCP-1 gene therapy markedly reduced the infiltration of macrophages but not that of T cells in the infarcted myocardium. Dewald *et al*. (10) also reported that the infiltration of macrophages, but not neutrophils, was suppressed and delayed in MCP-1-null mice when compared with that in wild-type mice. These findings indicate that MCP-1 is a critical factor for the recruitment of monocytes/macrophages in the infarcted heart.

### Myocardial healing and scar formation

Macrophages and myofibroblasts play a crucial role in the process of myocardial repair after MI. Macrophages phagocytose the necrotic myocardium and concomitantly, myofibroblasts proliferate and migrate into the infarcted area (2, 65). The necrotic tissue is replaced by the granulation tissue. As repair proceeds, myofibroblasts secrete and deposit collagen and other extracellular matrixes; subsequently, apoptosis of the granulation tissue cells results in the formation of a thin and hypocellular scar formation (66). Progressive cardiac wall thinning and chamber dilatation (remodeling) are associated with the increased incidence of congestive heart failure, aneurysm formation, and mortality.

MCP-1 may influence the replacement of granulation tissue and myofibroblast accumulation in the healing process after MI. MCP-1 directly modulates fibroblast activity by increasing collagen (67) and matrix metalloproteinase expression (68). Dewald *et al*. (10) reported that in the infarcted heart of murine ischemia-reperfusion models, MCP-1 deficiency delays macrophage recruitment and replacement of cardiomyocytes by the granulation tissue. They further demonstrated that after MI, MCP-1 deficiency diminished myofibroblast accumulation and attenuated left ventricular remodeling. We recently showed that MCP-1 directly promotes the *in vitro* differentiation of cardiac fibroblasts into myofibroblasts (8). In addition, the hypoxic conditions increased the differentiation of cardiac fibroblasts and it was further enhanced in the presence of MCP-1. In fact, after MI, myofibroblast accumulation had notably increased in the infarcted myocardium in MHC/MCP-1 mice in contrast to that observed in wild-type mice. Taken together, these findings suggest a critical role for MCP-1 in myocardial healing, scar formation, and remodeling after MI.

### Angiogenesis

Angiogenesis could affect infarct size and myocardial remodeling by supplying oxygen and nutrients necessary to maintain metabolism. Recent evidence indicates that MCP-1 promotes the *in vitro* and *in vivo* formation of new blood vessels in ischemic tissues. Weber *et al*. (58) reported that human endothelial cells express CCR2, which is upregulated by inflammatory cytokines, and MCP-1 induces migration and proliferation of the endothelial cells. After mechanical injury to the endothelial monolayers, which spontaneously closed within 24 hours, wound repair was delayed by a CCR2 antagonist and an MCP-1 blocking antibody. Similarly, using the chick chorioallantoic membrane (CAM) and matrigel plug assays, Salcedo *et al*. (57) showed that MCP-1 induces *in vivo* formation of new blood vessels. In addition, Schwarz *et al*. (69) tested the effects of MCP-1 on angiogenesis (newly developed capillaries) and arteriogenesis (the development of functionally active arterioles from pre-existing blood vessels) in a chronic MI rat model; they showed that an intramyocardial injection of recombinant MCP-1 into the infarct border zone induces monocyte infiltration and angiogenesis but not arteriogenesis in the infarcted heart. Recently, we also observed in MHC/MCP-1 mice that capillary formation and macrophage infiltration significantly increased after MI. Moreover, Low *et al*. (70) demonstrated that MCP-1^-/-^ mice exhibit delayed wound angiogenesis demonstrating lower capillary density than their wild-type littermates; this suggests an important role of this chemokine in wound healing and concurrent angiogenesis.

Several possible mechanisms have been postulated for the induction of angiogenesis by MCP-1. First, MCP-1 directly upregulates *HIF-1α* gene expression and subsequently stimulates VEGF induction by endothelial cells (71). MCP-1 has also been shown to stimulate VEGF induction by macrophage recruitment to the ischemic tissues (72). Second, recent evidence indicates that the bone marrow-derived monocytic lineage cells function as endothelial progenitor cells (EPCs) and participate in the formation of new blood vessels in the ischemic tissues. Our recent study showed that in contrast to wild-type mice, MHC/MCP-1 mice did not show an increase in the EPCs (CD34^+^/Flk-1^+^ cells; known as an ordinary EPC marker (73)) in the peripheral circulation after MI; this suggests that the monocytic cell-derived EPCs may have other surface markers. In this regard, Harraz *et al*. (74) reported that the CD34-negative angioblasts are a subset of the CD14-positive monocytic cells, and these monocytes have the potential to transdifferentiate into endothelial cells. Fujiyama *et al*. (75) reported that the bone marrow-derived CD34^-^/CD14^+^ EPCs adhere to the injured endothelium of a rat carotid artery in an MCP-1-dependent manner and accelerate endothelial repair; this suggests that MCP-1 may stimulate bone marrow-derived monocytic EPCs. Third, interesting findings by Moldovan and his colleagues (76) revealed that the monocytes/macrophages accumulated by MCP-1 produce matrix metalloproteinase-dependent tunnels and promote the formation of new vessels. In addition, more recently, they demonstrated that monocytes/macrophages are required to induce efficient angiogenesis and tissue repair besides inducing parent endothelial cells or EPCs (77). Taken together, MCP-1 regulates the process of angiogenesis in the infarcted myocardium, thereby influencing cardiac function and remodeling after MI.

## THERAPEUTIC IMPLICATIONS

The Framingham Heart Study demonstrated that variations in the MCP-1 gene influence serum MCP-1 levels and the incidence of myocardial infarction (6). Other clinical studies have also indicated that higher serum MCP-1 levels are associated with an increased risk of myocardial infarction, sudden cardiac death, coronary angioplasty, and stent restenosis (5, 78, 79). Thus, MCP-1 may be a potential target for therapeutic interventions in MI. Indeed, several experimental studies have suggested that the inhibition of MCP-1 signaling improves cardiac dysfunction and remodeling after MI (9, 11), suggesting a deleterious effect of MCP-1 in MI. In contrast, other investigators and we demonstrated that cardiac overexpression of MCP-1 improves cardiac dysfunction and remodeling after MI (8, 80). Furthermore, recent studies have shown that the role of MCP-1 extends beyond its monocyte chemoattractant effects; it also induces angiogenic and cardioprotective effects (57, 60, 61, 71). Since MCP-1 is produced in the infarcted heart (7, 81) and the effects of MCP-1 are regulated by its topical concentration (63, 64), the beneficial or deleterious effects of MCP-1 might depend on the situation, i.e. local concentration, duration, and time period after MI.

## CONCLUSIONS

A growing number of studies have indicated that chemokines are key mediators of MI. Of these, MCP-1 is one of the most important chemokines that is involved in the pathophysiology of MI. In this review, we discussed the role of MCP-1 in the processes of MI; myocardial necrosis and apoptosis, leukocyte recruitment, myocardial healing and scar formation, and angiogenesis. Further, MCP-1 is thought to be a potential target for the treatment of MI; however, prior to its clinical application of MCP-1, further investigations are necessary to elucidate its precise role in MI.
